# Screening, characterisation and prevention of Hepatitis B virus (HBV) co-infection in HIV-positive children in South Africa

**DOI:** 10.1016/j.jcv.2016.10.017

**Published:** 2016-12

**Authors:** Pieter Jooste, Anriette van Zyl, Emily Adland, Samantha Daniels, Louise Hattingh, Alethea Brits, Susan Wareing, Dominique Goedhals, Katie Jeffery, Monique Andersson, Philip Goulder, Philippa C. Matthews

**Affiliations:** a Department of Paediatrics, Kimberley Hospital, Kimberley, South Africa; b Department of Paediatrics, Peter Medawar Building, South Parks Road, Oxford OX1 3SY, UK; c Department of Infectious Diseases and Microbiology, Oxford University Hospitals NHS Foundation Trust, John Radcliffe Hospital, Headley Way, Headington, Oxford OX3 9DU, UK; d Department of Medical Microbiology and Virology, National Health Laboratory Service, University of the Free State, Bloemfontein, South Africa; e Nuffield Department of Medicine, Peter Medawar Building, South Parks Road, Oxford OX1 3SY, UK

**Keywords:** HBV, hepatitis B virus, WHO, World Health Organisation, EPI, expanded programme on immunisation, HIV, human immunodeficiency virus, HBsAg, hepatitis B surface antigen, HEU, HIV exposed uninfected, CDC, Centres for Disease Control, MPCI, magnetic parcel chemiluminometric immunoassay, HBeAg, hepatitis B e-antigen, HBeAb, hepatitis B e-antibody, Anti-HBc, IgM hepatitis B anti-core IgM antibody, HBsAb, hepatitis B surface antibody, IQR, interquartile range, Total Anti-HBc, total antibody to hepatitis B core protein, PCR, polymerase chain reaction, ABC, abacavir, 3TC, lamivudine, rLPV, ritonavir boosted lopinavir, TDF, tenofovir, FTC, emtricitabine, EFV, efavirenz, DTG, dolutegravir, Hepatitis B, Viral hepatitis, South Africa, Vaccination, Epidemiology, HIV

## Abstract

**Background:**

In South Africa, the first HBV vaccine dose is administered at age 6 weeks, leaving a potential window for vertical transmission. Insights into HBV seroprevalence in the vulnerable HIV-infected group are important to drive improvements in surveillance, treatment and prevention.

**Objectives:**

We set out to implement a screening program for HBV among HIV-infected children and adolescents in Kimberley, South Africa. Our aims were to demonstrate that screening is feasible and sustainable, to establish the prevalence of HBV, to characterise the HBV cases we identified, and to inform discussion about the infant vaccination schedule.

**Study design:**

We tested all HIV positive children (age 0–16) for Hepatitis B surface antigen (HBsAg), delivering this testing as part of routine state-funded care. We followed up HBsAg-positive cases with an extended panel of HBV serology tests, and HBV DNA viral load quantification.

**Results:**

Our screening campaign was successfully incorporated into routine out-patient care. Among 625 patients tested, we found five positive for HBsAg (0.8%), of whom three were Hepatitis B e-antigen positive. Two additional children initially tested HBsAg-positive but were negative on repeat testing. Antiviral therapy in the HBsAg children was reviewed and adjusted if required.

**Conclusions:**

The results testify to the overall success of the HBV vaccine campaign. However, we have demonstrated that ongoing vigilance is required to detect cases and prevent transmission events. Further evaluation of the optimum timing of the first vaccine HBV vaccine dose is required; a vaccine dose at birth could reduce prevalence further.

## 1 Background

Reflecting the success of vaccination campaigns, the global prevalence of chronic Hepatitis B Virus (HBV) infection has fallen since 1990; however, the absolute number of cases has continued to rise [1], highlighting the critical need for ongoing efforts to reduce transmission. The World Health Organisation Expanded Programme on Immunisation (EPI) recommends that all infants in settings where HBV prevalence is ≥2% should receive three or four doses of HBV vaccine, with the first dose in the first day of life [2].

HBV vaccination has been implemented in South Africa since 1995, but the locally recommended schedule is to postpone the first dose until age six weeks [3], [4]. Recently, HBV immunisation has been incorporated into a hexavalent vaccine together with diphtheria, tetanus, pertussis, inactivated poliomyelitis and *Haemophilus influenzae* type b (Hexaxim, Sanofi-Pasteur). It is recognized that children with HIV infection may not mount adequate vaccine-mediated immunity [5], potentially making this a vulnerable group.

In a recent study to ascertain the epidemiology of HBV in South African adults, we identified HBsAg in 8.3% of HIV-negative, and 9.7% of HIV-positive adults [6]. Published around the same time, a study from the Western Cape reports 0.4% prevalence of HBV in HIV exposed-uninfected (HEU) infants, all of whom were HBV-immunised according to local policy [4]. Together, these data highlight that chronic HBV is a persistent problem in South African adults, irrespective of HIV status, and that infants remain vulnerable to vertical acquisition of HBV infection before receiving their first dose of vaccine. In this setting, the high seroprevalence of HIV infection puts infants and young children at increased risk of co-infection and of complications of chronic HBV infection [4], [7]. Our recent study found HBsAg in 11% of HIV-positive mothers of children attending outpatient clinics in Kimberley [6], but HBV-screening has not been routinely incorporated into clinical care.

## 2 Objectives

We set out to investigate whether there is evidence of ongoing HBV transmission in our HIV-positive paediatric cohort in Kimberley, South Africa, to ascertain the feasibility of incorporating this into routine clinical care, and to characterise the cases of infection identified. This study aims to inform ongoing efforts in HBV screening, treatment and prevention.

## 3 Study design

We implemented screening for HBsAg in HIV-infected children (age <17 years) attending out-patient clinics at Kimberley Hospital in South African’s Northern Cape, during a fifteen-month period commencing March 2015. HBV screening was introduced as part of routine clinical care in keeping with WHO and CDC recommendations [8], [9].

Among children who tested HBsAg positive, we obtained written informed consent for study enrollment from the child (according to age/ability) and their parent/guardian. This study was approved under the terms of the Ethics Committee of the Faculty of Health Science, University of Free State, Bloemfontein, South Africa (ref. ETOVS Nr 08/09).

We recorded HBV vaccination history for children who attended with their hand-held records (Road to Health Book). HBsAg testing was performed by the clinical laboratory at Kimberley Hospital, using the Magnetic parcel chemiluminometric immunoassay (MPCI; Advia Centaur platform), re-testing samples from an alternative time-point to confirm, where possible. For children testing HBsAg positive, we stored serum at −80 °C and shipped samples to the Oxford University Hospitals microbiology laboratory. We repeated testing for HBsAg, and additionally performed assays for HBeAg, HBeAb, HBc IgM, HBsAb (all on Architect i2000), and HBV DNA quantification (Cobas ampliprep).

## 4 Results

Over a 15-month period, we screened 625 HIV-infected children and adolescents for HBV infection (331 male, 294 female; median age 10.8 years, IQR 6.6–13.8 years). We identified five (0.8%) who were positive for HBsAg (Table 1) including a sibling pair (KHep004 and KHep005).

A further two children who initially tested HBsAg-positive (KHep001 and KHep003) were negative on repeat testing, and also negative for anti-HBc and for HBV DNA, suggesting that the initial assay had yielded a false positive result. KHep006 followed the same initial pattern (initially HBsAg positive, but negative on follow-up) but had detectable HBV DNA, supporting the presence of true infection. For KHep007, our confirmation HBsAg test was done on a sample collected at an earlier timepoint (based on sample availability). This child tested negative in 2014, but positive in 2016, suggesting either fluctuations in low level HBsAg, or a new infection acquired subsequent to 2014. These cases collectively illustrate the importance of following-up positive screening results with a confirmation HBsAg test and PCR for HBV DNA.

Only 45 children in this cohort (7%) attended clinic with their vaccine records (Road to Health Book). Not surprisingly, this group was significantly younger than the rest of the cohort, as parents/guardians of infants and younger children are more likely to attend with vaccine records (median age of children with records 3.9 years, compared to 11.3 years in those without; p < 0.0001). Of these 44/45 had received all three HBV vaccine doses, and the remaining child had received two doses. One of these 45 was KHep003, who initially tested HBsAg positive, but was negative for HBsAg and HBV DNA on follow-up.

One of the five children with detectable HBV DNA had a positive anti-HBs result (KHep006), suggesting prior immunization. This child seroconverted from HBsAg-positive to HBsAg-negative, and had only low-level viraemia. We did not identify any child positive for anti-HBc IgM, suggesting that none of the five cases was acutely infected. We detected HBeAg in 3/5 HBsAg-positive children; no case had detectable anti-HBe (Table 1).

Current South African ART guidelines recommend children are treated with ABC + 3TC + either rLPV or EFV, but make no specific recommendations for therapy in the context of HBV co-infection [10]. At the time of screening positive for HBsAg, all five of our HBV-infected children were on ART regimens incorporating HBV-active agents (3TC or TDF). In the case of KHep002, the high HBV DNA level despite 3TC-containing therapy prompted a therapy switch. Efforts are currently underway to identify and screen household contacts with the view to treating infection and vaccinating susceptible individuals.

## 5 Discussion

This study demonstrates that HBV screening is feasible as part of routine clinical care for children and adolescents with HIV attending outpatient clinics in South Africa. We now propose to continue this screening as part of routine state-funded care.

Identifying HBV cases allows optimization of antiviral treatment regimens; the case of KHep002 highlights how diagnosing and monitoring HBV infection can have important bearing on the choice of ART regimen. The identification of an infected sibling pair in this cohort demonstrates the need for particular vigilance around the risk of clustering in families, underlining the importance of preventive measures in tandem with screening.

Both our data-set and another recent study [5] demonstrate the success of the existing vaccine campaign in reducing paediatric HBV prevalence. However, there is undoubtedly a need for ongoing vigilance and efforts to reduce transmission still further. Together with the recent results from the Western Cape [4], these studies suggest that strategies to prevent mother to child HBV transmission have been successful, but need to remain an important focus. Furthermore, there are ongoing challenges; HBV immunisation is not routinely available for adults in the public sector (though should be available for those at risk), and availability of the new hexavalent vaccine has been problematic.

Routine maternal screening for HBV during pregnancy would yield benefits for both mother and child, including clinical assessment and appropriate treatment for adult infection, and implementation of measures to reduce both vertical and within-household transmission [11]. Ongoing evaluation of the risks and benefits of the timing of the first HBV vaccine dose is required. HBV immunisation at birth would further reduce perinatal transmission events, and could be given as a monovalent vaccine, with the hexavalent vaccine following at six weeks to boost antibody responses.

The data presented here may under-represent the true prevalence of HBV infection, by missing cases with low level HBs-antigenaemia or in which mutations in HBsAg render the surface antigen assay negative [12]. In future studies, screening using HBV DNA as a more sensitive measure could be considered. However, this is not routinely available and is more expensive, and is therefore currently not feasible in South Africa.

We have been unable to make a robust assessment of the specific details of vaccination history within this cohort, as only 7% of the cohort had available vaccination records. This small subgroup cannot be deemed representative of the wider population (indeed, it would be reasonable to hypothesise that families most likely to access all three vaccine doses might also be those who are also most likely to bring their vaccine records to clinic).

Future studies are therefore required to quantify the titres of vaccine-mediated HBsAb present in this population. Nevertheless, a prevalence of HBV infection of <1% in children compared to 11% in the adult population [6] suggests a substantial reduction in transmission, much of which is likely to be attributable to successful vaccination. While celebrating the impact of campaigns to reduce HBV infection, we should maintain a high level of scrutiny to detect cases and continue intensive efforts to reduce transmission.

## Conflicts of interest

We have no conflicts of interest to declare.

## Funding

This work was supported by NIHR research capability funding (PM) and PG the Wellcome Trust, ref. WT 104748MA (PG). HBsAg testing was provided as part of routine clinical care at Kimberley Hospital. Sample transport and additional laboratory consumables for the additional panel of HBV tests were funded by grants from the Oxford University Medical Research Fund (PM) and the Rosetrees Trust (PM).

## Figures and Tables

**Table 1:** Characteristics of five HIV-infected children testing positive for HBsAg in Kimberley, South Africa.

Study ID^a^	KHep002 (ART2156)	KHep004^b^ (ART1917)	KHep005^b^ (ART2096)	KHep006 (ART1585)	KHep007 (ART1638)
Sex	F	M	F	M	F
Age (years) at time of HBV screening	6.8	6.4	15.6	5.6	5.3
Number of doses of HBV vaccine	3	Unknown	Unknown	Unknown	Unknown
HBsAg test date and result (MPCI^c^ Kimberley)	Oct 2015; Detected	Apr 2015; Detected	Jul 2015; Detected	Jul 2015; Detected	Jan 2016; Detected
Repeat HBsAg test date and result (MPCI^b^, Kimberley)	Nov 2015; Detected	Nov 2015; Detected	Nov 2015; Detected	Feb 2016; Detected	Not done
Date of sample used for confirmation HBsAg test; result (Architect, Oxford)	Nov 2015; Detected	Jan 2014; Detected	Nov 2015; Detected	Feb 2016; Not detected	Sept 2014; Not detected
Hepatic transaminase levels (ALT, AST)	Within normal reference range	Within normal reference range	Within normal reference range	Within normal reference range	Within normal reference range
Date and result of most recent HIV VL (RNA copies/ml)	Apr 2015; 10,931	Apr 2015; <40	01/08/2013; 196,111	05/01/2014; 25	01/01/2015; <150
Date and result of most recent CD4+ T cell count (CD4%)	Apr 2015; 566 (26%)	Apr 2015; 1500 (38.5%)	Feb 2016; 1883 (40.5%)	Sept 2015; 1135 (31%)	Jan 2015; 2012 (37.3%)
ART^d^: start date and regimen	Jun 2014; ABC/3TC/ EFV In 2016, switched to TDF/FTC/rLPV	Oct 2012; ABC/3TC/EFV	Jan 2014; EFV/FTC/TDF	Jan 2014 ABC/3TC/rLPV	Mar 2011; ABC/3TC/rLPV Since 2014, taking 3TC only^e^
HBV DNA (iu/ml)	36,830,065	136,875	110	260	167
HBeAg^f^	Detected	Detected	Detected	Not detected	Not detected
Anti-HBe^g^	Not detected	Not detected	Not detected	Not detected	Not detected
HBsAb^h^ (iU/L)	Not detected	Not detected	Not detected	Detected (212.2)	Not detected
Anti-Hbc IgM^i^	Not detected	Not detected	Not detected	Not detected	Not detected
Total AntiHBc^j^	Not detected	Not detected	Not detected	Not detected	Not detected

a Each child testing positive for HBsAg was assigned a new number with the prefix K-Hep (Kimberley Hepatitis). In addition, these children each have an ART (Anti-Retroviral Therapy) study number which we have retained to ensure we can continue to identify these children for clinical follow-up, and in order to permit cross-reference between studies.

b KHep004 and KHep005 are a sibling pair (same mother; different fathers; mother is deceased).

c MPCI – Magnetic Parcel Chemiluminometric Immunoassay.

d ART – antiretroviral therapy; abbreviations for drugs are as follows: ABC = abacavir; 3TC = lamivudine; rLPV = lopinavir with ritonavir boost (children on rLPV are taking combination in the form of Kaletra); EFV = efavirenz.

e KHep007 is on 3TC only at present due to difficulties with drug adherence, with a plan in place to continue close surveillance and ultimately restart triple therapy, potentially comprising FTC/TDF/DTG.

f HBeAg – Hepatitis B e-Antigen.

g Anti-HBe – Antibody to Hepatitis B e-Antigen.

h HBsAb – Hepatitis B surface antibody (vaccine mediated).

i Anti-Hbc IgM – IgM antibody to Hepatitis B core protein.

j Total Anti-HBc – Total antibody to Hepatitis B core protein.
